# Long-term follow-up of combination therapy with pembrolizumab and anlotinib in thoracic SMARCA4-deficient undifferentiated tumor: a case report and molecular features

**DOI:** 10.3389/fonc.2024.1453895

**Published:** 2024-12-11

**Authors:** Ting Duan, Mingxin Xu, Haibo Zhang, Shengchang Wu, Haochu Wang, Zhenying Guo

**Affiliations:** ^1^ Cancer Center, Department of Pathology, Zhejiang Provincial People’s Hospital(Affiliated People’s Hospital), Hangzhou Medical College, Hangzhou, China; ^2^ Department of Pathology, Tongxiang First People’s Hospital, Tongxiang, China; ^3^ Cancer Center, Department of Radiation Oncology, Zhejiang Provincial People’s Hospital(Affiliated People’s Hospital), Hangzhou Medical College, Hangzhou, China; ^4^ Cancer Center, Department of Pulmonary and Critical Care Medicine, Zhejiang Provincial People’s Hospital(Affiliated People’s Hospital), Hangzhou Medical College, Hangzhou, China; ^5^ Cancer Center, Department of Radiology, Zhejiang Provincial People’s Hospital (Affiliated People’s Hospital), Hangzhou Medical College, Hangzhou, China

**Keywords:** lung cancer, SMARCA4-UT, immune checkpoint inhibitors, anti-angiogenic agent, prognosis, genen mutation

## Abstract

Thoracic SMARCA4-deficient undifferentiated tumors (SMARCA4-UTs), recently recognized as a rare malignancy described in the 5th edition of the World Health Organization Classification of Tumors, are characterized by an inactivating mutation in SMARCA4, most commonly found in the mediastinum of male smokers. Despite the aggressive nature and poor prognosis associated with these tumors, which have a median survival time of approximately 4-7 months, no standardized treatment guidelines are currently established. There are currently no reported cases of extended progression-free survival (PFS) in SMARCA4-UT patients treated with surgery and immunotherapy. Here, we report the clinical features and genomic information of a SMARCA4-UT case in which the patient responded significantly to a combination therapy involving surgery, immunotherapy, and amlotinib. A 56-year-old male non-smoker presented with a mass in the superior lobe of left lung and left hilar adenopathy. A left upper lobectomy and lymphadenectomy were performed, and postoperative pathology confirmed that the tumor was Thoracic SMARCA4-UT. The patient subsequently received chemotherapy with pemetrexed and carboplatin. Five months post-operation, the disease progressed with left adrenal metastasis and mediastinal adenopathy. An adrenalectomy was performed, followed by whole exon sequencing (WES). SMARCA4, SMARCA2 and SMARCA1 gene mutations were detected in this case. Given a tumor proportion score (TPS) of 60% for programmed death-ligand 1(22C3)immunoexpression and high TMB(361.32 muts/Mb), a combination of Pembrolizumab plus anlotinib was initiated as a second-line approach. After 46 cycles, the patient demonstrated no disease progression with a PR lasting 31 months and long progression-free survival(PFS) of 43 months. The lung tumor was initially detected in September 2020, and the patient remained alive at the latest follow-up in November 2024. This case offers a long-term follow-up of the effectiveness and safety of combining pembrolizumab and anlotinib in advanced SMARCA4-UT, and substantiates the role of long-term immunotherapy in preventing radiographic/clinical recurrence following surgery. This case illustrates new potential efficacy of immunotherapy in combination with surgery as a treatment approach of SMARCA4-UT.

## Introduction

Thoracic SMARCA4-deficient undifferentiated tumor (SMARCA4-UT) was first recognized as a distinct entity in the 5th edition of the 2021 World Health Organization Classification of Tumors (1). It is a high-grade malignancy that commonly occur in the mediastinum, lung, pulmonary hilum, and pleura. SMARCA4-UT often affects young to middle-aged adults with heavy smoking history and a striking male predominance (2–5). Effective treatments have not yet been developed, and the prognosis remains poor, with a median survival of 4-7 (range, 1-13)months (2). Due to the high probability of early recurrence, upfront surgery is not an effective treatment (6). Although radiotherapy and chemotherapy have limited efficacy, immune checkpoint inhibitors (ICIs) have shown promising results in some patients (7–12). However, given the limited number of patients and short survival duration during the follow-up, rare long-term follow-up cases have been reported in advanced SMARCA4-UT patients. Herein, we report a case of Thoracic SMARCA4-UT with adrenal metastasis, who underwent surgical resections and was successfully treated with Pembrolizumab plus anlotinib, achieving a PR lasting 31 months and long progression-free survival (PFS) of 43 months.

## Case description

**Figure 1:**
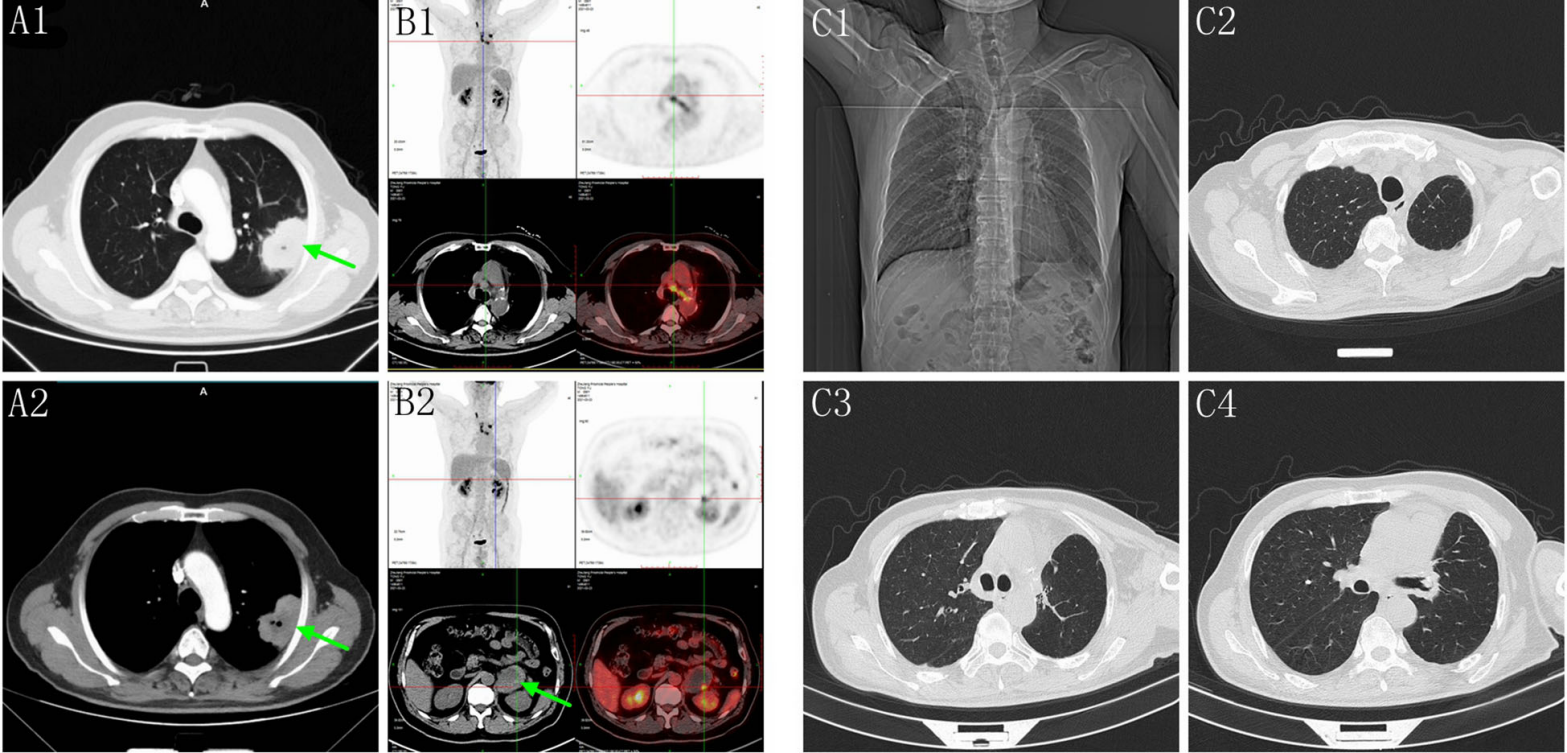
**(A1, A2)** Chest CT showed a solid mass in the superior lobe of left lung (green arrow). **(B1, B2)** Five months post-surgery, PET-CT revealed mediastinal adenopathy and a circular mass in the left adrenal gland measuring 8.5x5.6x4.3 cm (green arrow). **(C1–C4)** Chest CT showed no recurrence and the mediastinal lymph nodes were significantly reduced on September 26, 2024.

**Figure 2:**
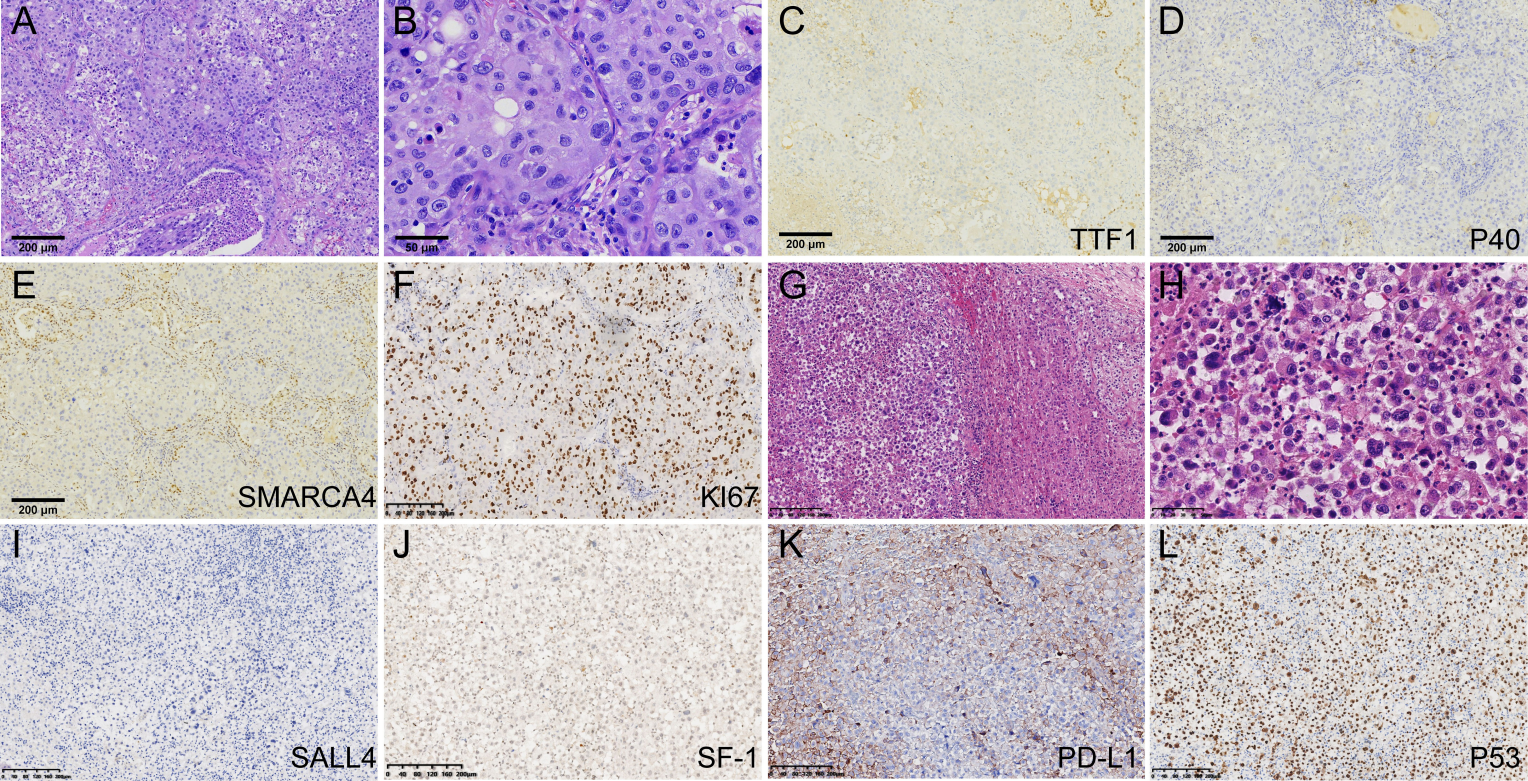
Pathological findings and immunohistochemical results. **(A)** The lung tumor cells arranged in sheets and were surrounded by an infiltration of inflammatory cells(HE100). **(B)** Large tumor cells showed obvious atypia, and prominent nucleoli(HE400). **(C)** TTF-1 staining was negative(x100). **(D)** P40 staining was negative(100). **(E)** SMARCA4 staining was negative in the tumor cells(100). **(F)** ki67 was as high as 70%. **(G, H)** The tumor cells of left adrenal gland were poorly differentiated, displaying discohesion, marked atypia and prominent nucleoli. **(I)** SALL4 staining was negative. **(J)** SF-1 was negative in the tumor cells. **(K)** PD-L1 TPS score was 60%. **(L)** P53 is overexpressed.

A 56-year-old male, non-smoker, presented with recurrent low fever in September 2020 with no prior history of tumors. Chest computed tomography (CT) revealed a solid mass in the superior lobe of left lung adjacent the pleura, measuring 5.5x5.0x4.0 cm, accompanied by left hilar adenopathy (**Figure 1A**). PET-CT showed no bone and distant metastasis. Comprehensive laboratory tests were within normal limits, and blood tests showed no abnormal squamous cell carcinoma antigen (SCC), carcinoembryonic antigen (CEA), or neuron-specific enolase (NSE) levels. On October 10, 2020, the patient underwent a left upper lobectomy and lymphadenectomy. Postoperative pathology revealed a solid tumor structure with large epithelioid cells, obvious atypia, and prominent nucleoli (**Figures 2A, B**). Immunohistochemistry was positive for SMARCB1/INI1, focally positive for cytokeratin and negative for TTF-1 (**Figure 2C**), NapsinA, P40 (**Figure 2D**), SMARCA4 (**Figure 2E**), CK5/6, Nut, Vimentin, EBER. Ki-67 was as high as 70% (**Figure 2F**). These findings led to a diagnosis of Thoracic SMARCA4-UT, classified as stage IIIA (pT3N1M0).

Subsequently, the patient received chemotherapy (pemetrexed approximately 960 mg day 1 and carboplatin approximately 670 mg day 1 every 4 weeks) with Neulasta support for sustained leukocyte enhancement from December 5, 2020 to March 6, 2021. Blood tests during chemotherapy showed white blood cells at 4.4x10^9/L, hemoglobin at 97 g/L, neutrophil count at 1.9x10^9/L, and platelet count at 245x10^9/L, albumin at 36.7 g/L, ALT at 38 U/L, AST at 28 U/L, creatinine at 87 μmol/L, and urea at 7.48 mmol/L. The patient developed mild anemia and minor adverse reactions throughout the course of chemotherapy.

Five months post-surgery, PET-CT revealed mediastinal adenopathy suggesting recurrence, and a circular mass in the left adrenal gland measuring 8.5x5.6x4.3 cm on March 23, 2021 (**Figure 1B**). No anomalies were detected in the contralateral adrenal gland. Left adrenalectomy was performed on March 26, 2021. Histologically, the tumor cells were poorly differentiated, displaying discohesion, marked atypia and prominent nucleoli (**Figures 2G, H**). Immunohistochemistry was positive for INI1, and negative for SMARCA4, TTF-1, NapsinA, NUT, SALL4 (**Figure 2I**), and SF-1 (**Figure 2J**). PD-L1 (clone 22C3) tumor proportion score (TPS) was 60% (**Figure 2K**), and P53 is overexpressed (**Figure 2L**). DNA were extracted from paraffin embedded tumor tissues of the left adrenal gland, and underwent whole-exome sequencing (WES) (AZENTA Life Science [Suzhou] Co. Ltd, Suzhou China). The sequencing results revealed a frameshift deletion in SMARCA4 gene p.R1009fs (c.3027delC), a nonsynonymous SNP in SMARCA2 gene p.V815I (c.G2443A) and a nonsynonymous SNV in SMARCA1 gene p.A269V (c.C806T). This was along with mutations in KRAS (p.G12A), LRP1B (p.C2739Y), ARID2 (p.G803D) and TP53. WES data analysis showed that the tumor mutation burden (TMB) was 361.32 muts/Mb. The genomic profile is demonstrated in **Table 1**. Consequently, the final diagnosis was SMARCA4-UT with adrenal metastasis, classified as Stage IV rT0N1M1b.

**Table 1:** Genetic alterations of Patient.

Chrom	Gene	CytoBand	Transcript Variant	Protein Variant	Mutation Type	Allele Frequency (%)
1	SETDB1	1q21.3	NM_001145415:exon11:c.1301_1304del	p.Y434fs	frameshift_deletion	6.2
1	PARP1	1q42.12	NM_001618:exon21:c.G2831A	p.G944D	nonsynonymous_SNV	2.2
1	PARP1	1q42.12	NM_001618:exon9:c.G1286A	p.C429Y	nonsynonymous_SNV	1.3
2	HOXD8	2q31.1	NM_001199746:exon1:c.51_52insGC	p.A17delinsAA	nonframeshift_insertion	51.9
2	LRP1B	2q22.1	NM_018557:exon51:c.G8216A	p.C2739Y	nonsynonymous_SNV	7
3	PBRM1	3p21.1	NM_001366070:exon16:c.1649+3G>A; NM_001366076:exon15:c.1544+3G>A; NM_001350077:exon18:c.1640+3G>A		splicing	1.1
3	MUC4	3q29	NM_018406:exon2:c.11209_11210insTATCTACAGGTCACGTCACCCCTCTTCATGTCACCAGCCCTTCCTCAG	p.A3737delinsVSTGHVTPLHVTSPSSA	nonframeshift_insertion	4
4	BMP2K	4q21.21	NM_017593:exon1:c.71_72insCGGGGC	p.G24delinsGGA	nonframeshift_insertion	46.7
4	TET2	4q24	NM_001127208:exon3:c.1527delA; NM_001127208:exon11:c.C4931T	p.S509fs; p.P1644L	frameshift_deletion; nonsynonymous_SNV	7
4	DCHS2	4q31.3	NM_001358235:exon20:c.8660dupT	p.F2887fs	frameshift_insertion	44.6
5	LIFR	5p13.1	NM_001127671:exon2:c.44_45insCGTATGTTTGAAACGACCATCCTGGATGGT	p.V15delinsVVCLKRPSWMV	nonframeshift_insertion	1.2
6	SYNCRIP	6q14.3	NM_001159675:exon10:c.1626_1627insGGTACAACCAGCCAGATTCCAAGCGG; NM_006372:exon11:c.1731_1732insGGTACAACCAGCCAGATTCCAAGCGG	p.R543fs; p.R578fs	frameshift_insertion	1.5
7	TRRAP	7q22.1	NM_003496:exon66:c.G10371A; NM_001244580:exon67:c.G10458A	p.M3457I; p.M3486I	nonsynonymous_SNV	4.8
9	SMARCA2	9p24.3	NM_001289396:exon17:c.G2443A	p.V815I	nonsynonymous_SNV	1.1
10	CSTF2T	10q21.1	NM_015235:exon1:c.G1006A	p.D336N	nonsynonymous_SNV	1.3
11	CD6	11q12.2	NM_001254750:exon7:c.1250_1251insCTTCATAGCCTTCATCCTCTTGAGAATTAAAGGAAAATATGGTAAGTGCAAGGTTCTGGGAGC	p.I417delinsIFIAFILLRIKGKYGKCKVLGA	nonframeshift_insertion	3.6
11	ATM	11q22.3	exon46:c.G6680A	p.R2227H	nonsynonymous_SNV	0.8
12	ARID2	12q12	NM_001347839:exon15:c.G2408A	p.G803D	nonsynonymous_SNV	1.1
12	GPRC5D	12p13.1	NM_018654:exon1:c.322_335del	p.L108fs	frameshift_deletion	0.7
12	TDG	12q23.3	NM_001363612:exon7:c.535_536insAGGATGCAAAGAAGATGGCTGTTAA; NM_003211:exon8:exon8:c.964_965insAGGATGCAAAGAAGATGGCTGTTAA; NM_001363612:exon8:c.661_662insTTGAGAGC;NM_003211:exon9:c.1090_1091insTTGAGAGC	p.E179fs;p.E322fs; p.I221fs; p.I364fs	frameshift_insertion	11.5
12	NCOR2	12q24.31	NM_001077261:exon40:c.6192delC; NM_006312:exon41:c.6222delC	p.L2064fs; p.L2074fs	frameshift_deletion	28.6
12	KRAS	12p12.1	NM_001369786:exon2:c.G35C	p.G12A	nonsynonymous_SNV	9.5
15	DLL4	15q15.1	NM_019074:exon4:c.598_599insGCCATCTGGCTGGCACACATAGTG	p.C200delinsCHLAGTHSG	nonframeshift_insertion	2.1
15	SIN3A	15q24.2	NM_001145357:exon21:c.3728_3729insACAGGGCACCAG	p.V1243delinsVQGTR	nonframeshift_inse	1.4
17	SLC9A3R1	17q25.1	exon5:c.799_799del	p.E267fs	frameshift_deletion	48
17	TSEN54	17q25.1	NM_207346:exon8:c.G1203A	p.W401X	stopgain	23.5
17	TP53	17p13.1	NM_001126115:exon3:c.G347A;NM_001276697:exon3:c.G266A; NM_001126118:exon6:c.G626A; NM_000546:exon7:c.G743A	p.R116Q; p.R89Q; p.R209Q; p.R248Q;	nonsynonymous_SNV	8
19	DAZAP1	19p13.3	NM_001352035:exon9:c.264_268del; NM_001352033:exon10:c.780_784del; NM_001352034:exon10:c.783_787del	p.P88fs; p.P260fs; p.P261fs	frameshift_deletion	6
19	SMARCA4	19p13.2	NM_001128845:exon20:c.3027delC	p.R1009fs	frameshift_deletion	12
19	RAVER1	19p13.2	NM_001366174:exon7:c.1251_1254del	p.E417fs	frameshift_deletion	15.4
19	DOT1L	19p13.3	NM_032482:exon13:c.1092_1093del	p.V364fs	frameshift_deletion	16.7
19	AMH	19p13.3	NM_000479:exon4:c.811delT	p.F271fs	frameshift_deletion	16.7
19	ERCC2	19q13.32	NM_001130867:exon9:c.868delG; NM_000400:exon10:c.940delG	p.V290fs; p.V314fs	frameshift_deletion	18.2
20	CHGB	20p12.3	exon4:c.1338_1340del	p.446_447del	nonframeshift_deletion	1.9
20	LAMA5	20q13.33	NM_005560:exon72:c.9882delC	p.P3294fs	frameshift_deletion	14.3
X	SMARCA1	Xq25	NM_001282874:exon6:c.C806T	p.A269V	nonsynonymous_SNV	2.3

DNA were extracted from the resected formalin fixed and paraffin embedded tumor tissues of the adrenal gland, and subjected to whole-exome sequencing(WES). Germline mutations were excluded with the use of the Human Genetic Variation Database (http://www.genome.med.kyoto-u.ac.jp/SnpDB) and the Exome Aggregation Consortium database.

Due to high PD-L1 expression and TMB, ICIs plus antiangiogenic agents were initiated as second-line treatment (pembrolizumab 200 mg D1, anlotinib 10 mg qd D1-D14 every 3 weeks) from April 2021 to October 2024. The patient was regularly monitored during treatment, and no evidence of disease progression was observed on chest and abdominal CT, brain MR scans. After 14 cycles of combined therapy, the patient had a partial response (PR), and chest CT showed a significant reduction of mediastinal lymph nodes on April 14, 2022. No severe toxicity or delayed toxicity occurred during treatment. Subsequently, the patient successfully completed 32 cycles of combined therapy, consisting of pembrolizumab 200 mg Day 1 and anlotinib 10 mg qd Day 1 to Day 14, repeated every 3 weeks as a sequential treatment. A recent chest CT showed no recurrence on September 26, 2024, with a significant reduction in mediastinal lymph nodes (**Figure 1C**).

**Figure 3:**
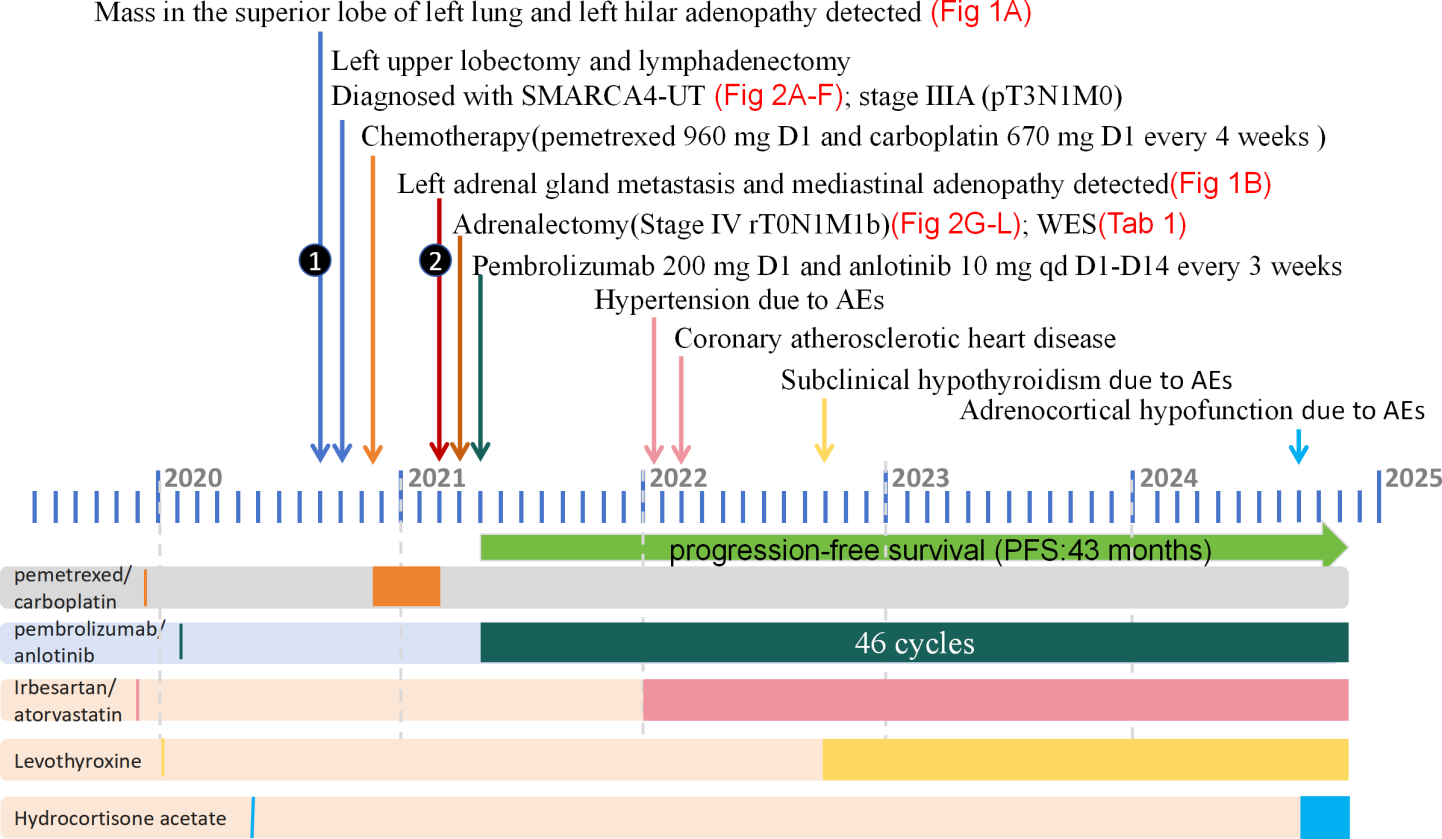
The diagnosis and treatment process of this case from September 2020 to November 2024. Surgery, chemotherapy, and immunotherapy are all represented in the upper part of the figure, with different colors representing different treatment modalities displayed in the lower part of the panel. SMARCA4-UT, SMARCA4-deficient undifferentiated tumor; WES, whole-exome sequencing; AE, adverse event.

The patients exhibited favorable tolerance towards the combination therapy. During the first month, the patient had mild pruritus and rash. After 8 months, the patient developed hypertension, managed with irbesartan. After 9 months, the patient felt chest tightness, and was diagnosed with coronary atherosclerotic heart disease and unstable angina pectoris. Coronary artery stenting was performed. After 16 months, the patient had subclinical hypothyroidism and was treated with levothyroxine. After 41 months, the patient developed adrenocortical hypofunction and was given hydrocortisone acetate tablets. Other clinical assessments, including body weight, blood and urine routine, hepatic and renal function, blood glucose and electrocardiograms, remained within normal limits. These side effects were classified as grade II according to CTCAE 5.0, and the patient did not have any serious immune-related side effects. The patient successfully completed 46 cycles of pembrolizumab combined with anlotinib and achieved a PR duration of 31 months and long PFS of 43 months at time of this report. The patient remained alive at the latest follow-up in November 2024. A summary of the patient's clinicial course is provided in **Figure 3**.

Written informed consent was obtained from the patient for the publication of all clinical data and images.

## Discussion

SMARCA4, located on chromosome 19p13, encodes for BRG1, one of the two ATPase subunits of the SWI/SNF (BRG1/BRM associated factor or BAF) complex, vital for chromatin remodeling and genomic regulation (5, 13). Inactivation of SMARCA4 (BRG1) has been suggested to be involved in the pathogenesis of some undifferentiated carcinomas of the lung, ovary, gastrointestinal tract, uterus, and other organs (14). Histologically, thoracic SMARCA4-UT typically have solid growth patterns with sheets of variably discohesive high-grade undifferentiated cells with eosinophilic cytoplasm, numerous mitoses, and prominent nucleoli (1–3). Most cases also exhibit a variable degree of rhabdoid morphology and necrosis (3). Immunohistochemically, SMARCA4-UT have a simultaneous deficiency of SMARCA4 (BRG1) and SMARCA2 (BRM) (5). Next-generation sequencing (NGS) are beneficial for the diagnosis. A review of recent literature indicates that the genomic mechanisms leading to SMARCA4 inactivation are variable, including mainly nonsense and frameshift mutations, with missense mutations, splice-site mutations, or deletions being less common (2, 3). Differential diagnoses for SMARCA4-UT include NUT carcinoma, lymphoma, mediastinal germ-cell tumor, round cell sarcomas, and neuroendocrine carcinoma, as well as various types of sarcomas (15).

Common metastatic sites for SMARCA4-UT include lymph nodes, bone, adrenal glands, and liver. A recent literature review of 102 documented cases with variable details revealed that adrenal involvement was present in 14 (14%) of the cases, while 20 (20%) exhibited other forms of intra-abdominal disease, most frequently affecting the liver and peritoneum (3). Coexisting bulky abdominal disease may make determination of primary site difficult (5). In clinical practice, the presence of a large adrenal mass may prompt the clinical consideration of an adrenal primary tumor, especially when the rhabdoid morphology may closely mimic an oncocytic adrenocortical carcinoma. Steroidogenic factor-1 (SF-1) is considered one of the most useful markers for distinguishing adrenal cortical lineage from other tumors (16). While one might consider the possibility of primary adrenal SMARCA4-deficient undifferentiated tumors, comprehensive genomic characterization and immunohistochemical feature of adrenocortical carcinomas have not revealed alterations in the SMARCA4 gene (17). Given the presence of a lung mass with left hilar adenopathy and considering the overall pathological and genetic characteristics of this case, negative expression of SF-1, it was concluded that the patient had a primary thoracic SMARCA4-UT with metastasis to the adrenal gland. This determination underscores the importance of a thorough evaluation of the tumor’s characteristics and metastatic patterns to accurately identify the primary site and guide treatment strategies.

SMARCA4-UT is a rare, highly aggressive malignancy, with limited response to chemotherapy and surgery. All patients essentially have disease progression or recurrence, and the cause of death was local complications due to disease burden (18). Recent studies have reported that ICIs show promising results in the treatment of SMARCA4-UT (7–12). Consensus predictors of immunotherapy efficacy include PD-L1 expression levels, TMB and MSI status. However, based on a review of previously published cases in SMARCA4-UT that had some response to ICIs, PD-L1 ranges from 0 to 100% (7–12). Thus, immunotherapy efficacy seems to be independent of PD-L1 expression. In the present case, PD-L1 TPS score was 60%, and WES TMB is 361.32 muts/Mb, greater than 199 muts/Mb, belonging to high TMB (19). In many cancer types, higher TMB was associated with poorer survival, in contrast to ICI-treated patients in whom higher TMB was associated with longer survival (20). Therefore, high TMB may be a valid indicator of immunotherapy in SMARCA4-UT. The biological mechanisms driving antitumor activity of ICI in SMARCA4-UT remains unclear, and further studies are required to identify predictive biomarkers and patients who will benefit from immunotherapy.

KEAP1, STK11, ARID1A, KRAS, and NF1 mutations are the second most common mutations in SMARCA4-UT (2). KEAP1 and STK11 mutations have been identified as associated with immunotherapy resistance in lung cancer (21). In this case, KRAS (p.G12A) mutation was found, but no KEAP1 or STK11 mutation were found. Studies have shown that KRAS mutation is associated with worse overall survival in PD-L1 negative NSCLC, and this association is largely driven by comutations with STK11 and KEAP1, which are enriched in PD-L1 negative tumors (22). Moreover, LRP1B and DNA damage repair genes (PARP1/2, ERCC1-4, MSH2-6, ATM, BRCA1/2, etc.) mutations have been shown to be associated with favorable outcomes to ICIs in lung cancer (23, 24). We also found LRP1B (p.C2739Y), ERCC2 (p.V290fs, pV314FS), PARP1 (p.Y434fs, p.G944D) and ATM (p.R2227H) mutations in this case. Therefore, these genetic characteristics may account for the good response to immunotherapy. We detected a nonsynonymous SNP in SMARCA2 gene p.V815I (c.G2443A) and a nonsynonymous SNV in SMARCA1 gene p.A269V (c.C806T), which are not registered in the Catalogue Of Somatic Mutations In Cancer database (25), and their oncogenicity is unknown.

## Conclusions

Herein, we report a case of 4-year long-term survival in a patient with Thoracic SMARCA4-UT and adrenal metastases who underwent surgeries and a combination therapy of pembrolizumab and anlotinib, achieving a PR duration of 31 months and PFS of 43 months. The lung tumor was initially detected in September 2020, and the patient remained alive at the latest follow-up in November 2024. Our case provides a long-term follow-up of the efficacy and safety of immunotherapy in advanced SMARCA4-UT, and highlights the potential of long-term immunotherapy in preventing radiographic/clinical recurrence after surgery. This illustrates new potential of immunotherapy in combination with surgery as an effective therapy in the treatment of SMARCA4-UT, and imaging changes can evaluate efficacy and long-term prognosis in these patients. Clinical trials exploring the role of ICI monotherapy or the combination of ICIs with other pathway targeting in SMARCA4-UT are needed, as are studies focused on predictive biomarkers and patient selection.

## Data Availability

The data presented in the study are deposited in the SRA database in NCBI, accession number PRJNA119820. The direct link is https://www.ncbi.nlm.nih.gov/sra.
